# ADAPTA: A pilot randomised controlled trial of an alcohol-focused intervention versus a healthy living intervention for problem drinkers identified in a general hospital setting^☆^

**DOI:** 10.1016/j.drugalcdep.2015.06.030

**Published:** 2015-09-01

**Authors:** Judith M. Watson, Caroline Fairhurst, Jinshuo Li, Gillian Tober, Helen Crosby, Charlie Lloyd, Christine Godfrey, Noreen D. Mdege, Veronica Dale, Paul Toner, Steve Parrott, Duncan Raistrick, On behalf of the ARiAS Research Group

**Affiliations:** NIHR CLAHRC for Leeds York Bradford; a University of York, Department of Health Sciences, Heslington, York YO10 5DD, United Kingdom; b Leeds Addiction Unit, 19 Springfield Mount, Leeds LS2 9NG, United Kingdom

**Keywords:** Alcohol-related hospital admissions, Healthy living, Acceptability, Problem drinkers, Social behaviour and network therapy, Randomised controlled trial

## Abstract

**Aim:**

To examine the relative feasibility, acceptability, applicability, effectiveness and explore cost-effectiveness of a healthy living focused intervention (HL) compared to an alcohol-focused intervention (AF) for problem drinkers identified in hospital.

**Methods:**

A pragmatic, randomised, controlled, open pilot trial. Feasibility and acceptability were measured by recruitment, attrition, follow-up rates and number of treatment sessions attended. Effectiveness was measured using the Alcohol Use Disorders Identification Test score at six months. Additional economic and secondary outcome measures were collected.

**Results:**

Eighty-six participants were randomised and 72% (*n* = 62) were retained in full participation. Forty-one participants attended at least one treatment session (48%). A greater proportion in the HL group attended all four treatment sessions (33% vs 19%). Follow-up rates were 29% at six months and 22% at twelve months. There was no evidence of a difference in AUDIT score between treatment groups at six months. Mean cost of health care and social services, policing and the criminal justice system use decreased while EQ-5D scores indicated minor improvement in both arms. However, this pilot trial was not powered to detect differences in either measure between groups.

**Conclusions:**

While no treatment effect was observed, this study demonstrated a potential to engage patients drinking at harmful or dependent levels in a healthy living intervention. However, recruitment proved challenging and follow-up rates were poor. Better ways need to be found to help these patients recognise the harms associated with their drinking and overcome the evident barriers to their engagement with specialist treatment.

## Highlights

• No evidence of a difference in AUDIT score was seen between treatments at 6 months.
• A greater proportion in the healthy living group attended all 4 treatment sessions.
• Recruitment and follow up proved challenging with this non-help seeking group.
• Further thought needed regarding engaging problem drinkers in a hospital setting.

## 1 Introduction

Alcohol is a contributing factor to over 60 types of disease and injury (Bendtsen and Åkerlind, 1999). The provision of specialist treatment for problem drinkers can reduce health-care resources use and costs to the public sector, resulting in considerable individual health and social benefits (Raistrick et al., 2006; Fleming et al., 1997, 2000). The majority of people admitted to hospital with alcohol-related illness are not seeking help regarding their drinking, nor are they identified or referred by hospital staff for specialist addiction treatment (World Health Organization, 2011; Madras et al., 2009). With alcohol-related hospital admissions rising (Jones et al., 2008), there is an opportunity for identification and treatment and there are recommendations that screening for alcohol problems form part of routine admission (Royal College of Physicians, 2001).

Previous studies have shown that the role of alcohol in disease and injury is often overlooked when it is not the presenting problem (Fahy et al., 2011; Raistrick et al., 2008). Hospital staff are reluctant to use admission as an opportunity to intervene, even where alcohol is a contributory factor to the admission (Raistrick et al., 2008). Discussing alcohol habits involves patients being asked to reveal potentially sensitive information about their drinking and research suggests that intervention engagement and acceptability are influenced by various factors, with a key issue being how such questioning is undertaken (Karlsson and Bendtsen, 2005), not least the attitudes and style of staff (Lock, 2004).

With lifestyles under scrutiny to an extent where unhealthy eating practices and smoking have been shown to produce stigmatised identities, addiction has been particularly pathologised (Bailey, 2005). Patients may be therefore reluctant to reveal their consumptive practices for fear of judgement. As such the acceptability of interventions can be argued to be a central feature in the effectiveness of interventions. Alcohol use, however, occurs in the context of overall health lifestyle (Bradstock et al., 1988) and a number of studies have suggested that health behaviours tend to cluster together among individuals (Schuit et al., 2002; Laaksonen et al., 2002). The notion that a healthy or balanced lifestyle is potentially beneficial for reducing alcohol use, abstaining from alcohol and for relapse prevention has been suggested (Marlatt and Gordon, 1985; Marlatt and Witkiewitz, 2005). The effect of a multi-factorial lifestyle approach on long-term alcohol behaviours in a general population was demonstrated by one population-based, pre-randomised, controlled study (Toft et al., 2009). However, despite this, healthy lifestyle approaches have received little emphasis in alcohol treatment literature (Brown et al., 2009).

This paper reports the outcomes from a pilot trial that explored the feasibility, acceptability, applicability, effectiveness and cost-effectiveness of a healthy living focused intervention compared to an alcohol-focused intervention, targeting hospital patients who have alcohol-related admissions but are not help seekers for treatment of a drinking problem. The terminology surrounding ‘problem drinking’ has been confusing and defined in various ways (Conigliaro et al., 2000). For this study we elected to define those scoring 16 or more on the Alcohol Use Disorders Identification Test (AUDIT; Babor et al., 2001) as ‘problem drinkers’. This includes harmful users where unhealthy alcohol use is already causing damage to physical or mental health and also dependent users where the level and pattern of use has resulted in a dependence syndrome which is a cluster of cognitive, behavioural and physiological symptoms (World Health Organization, 1993).

The hypothesis was that a healthy living intervention would have greater acceptability and patient adherence, and thereby better outcomes, than a specific alcohol intervention in terms of drinking behaviour change in this population. The pilot trial was built on earlier work, which explored hospital level patterns of admissions in three large general hospitals (part of a single National Health Service (NHS) Trust), current patterns of care and patient–care staff interactions (Sweetman et al., 2013; Mdege et al., 2014; Raistrick et al., 2014; Nazari and Raistrick, 2014).

## 2 Materials and methods

### 2.1 Design

Addressing Drinking Among Patients: comparing Two Approaches (ADAPTA) was a pragmatic, randomised, controlled, open pilot trial comparing a healthy living intervention with an alcohol-focused intervention for problem drinkers identified in a general hospital setting. The design has been reported in full elsewhere (Watson et al., 2013). The study was granted ethical approval by the National Research Ethics Service Committee Yorkshire and The Humber—Leeds Central (Reference: 11/YH/0448).

### 2.2 Participants

Participants were recruited from wards identified with the highest admission rates for patients with alcohol-related diagnoses in three general hospitals in England, UK. A Hospital In-Reach Team (HIRT) from a specialist addiction service visited the wards daily to try to engage patients in treatment, using a motivational interviewing style of consultation, in the same way as usual regardless of the research conditions. Patients with an alcohol-related diagnosis or a reason for admission that suggested a possible alcohol-related diagnosis (for example: pancreatitis, hypertension) were screened to determine eligibility as described previously (Watson et al., 2013), including a score of 16 or higher on the AUDIT (Babor et al., 2001).

### 2.3 The interventions

The alcohol focused (AF) intervention was based on the principles of social behaviour and network therapy (SBNT), an effective and cost-effective behaviour change intervention for help seeking problem drinkers (UKATT Research Team, 2005a, 2005b) involving a network of people who actively support positive change specifically in drinking. In the healthy living (HL) intervention, (based upon principles of behaviour change counselling with involvement of a supportive concerned other, or ‘buddy’, where one was available), the participant could choose to change their behaviour in up to three health behaviour domains from a choice of seven (drinking, drug use, diet, smoking, exercise, personal care and medication compliance). Both interventions involved prompting intention formation through encouraging participants to set a drinking goal or make a behavioural resolution, set interim goals and self-monitor behaviour change using a diary. Feedback on performance and review of behavioural goals was also provided.

Each intervention consisted of four 30–45 min sessions, which were ideally delivered one to two weeks apart and were intended to be completed over a maximum of eight weeks. Sessions could be delivered at the specialist clinic, the participants’ homes or at a mutually agreed alternative location as was current practice for the HIRT. All treatment sessions were to be recorded digitally for verification of intervention fidelity. Detailed descriptions of both interventions are reported elsewhere (Watson et al., 2013).

### 2.4 Outcome measures

Feasibility and acceptability were measured by recruitment and attrition rates, number of sessions attended and return rates of postal follow-up questionnaires. A range of outcome measures were employed to capture changes in substance use, dependence, therapeutic alliance and social and psychological functioning. Outcomes were assessed at six and twelve months using the following instruments: AUDIT (Saunders et al., 1993); Alcohol, Smoking and Substance Involvement Screening Test (ASSIST; WHO ASSIST Working Group, 2002); Leeds Dependence Questionnaire (LDQ; Raistrick et al., 1994); Social Satisfaction Questionnaire (SSQ; Raistrick et al., 2007); Clinical Outcomes in Routine Evaluation (CORE-10; Connell and Barkham, 2007); and the 12-item (short version) Working Alliance Inventory (WAI; Hatcher and Gillaspy, 2006).

### 2.5 Sample size

No formal power calculations were undertaken for this pilot trial. Based on caseload numbers, it was originally estimated that the maximum number of participants that could be recruited into the trial was 222 (111 in each group) over eight months. The post-treatment follow up rate was expected to be 70%, resulting in approximately 77 patients in each group, which would have given a detectable effect size difference of 0.45 on the AUDIT score at six months (80% power; two sided 5% significance).

### 2.6 Randomisation

Six therapists were divided into three pairs, matched on experience and qualifications. One of each pair delivered one of the treatments and received preliminary training followed by regular supervision of recorded practice. Participants were randomised to a treatment arm in a one-to-one ratio using the secure remote randomisation service at York Trials Unit, University of York. The participants’ allocated treatment intervention was delivered either by the therapist who screened them (to ensure continuity of care where possible) or their matched colleague. Where one of the therapists in the pair was not available, due to illness or otherwise, the participant was allocated to a therapist within a different pair. On a couple of occasions, participants who required home visits were seen by a specific therapist trained in the allocated intervention. Due to the nature of the interventions, it was not possible to blind participants or practitioners to their treatment allocation.

### 2.7 Statistical analysis

Analysis was performed in STATA v13, on an intention-to-treat basis using a two-sided 5% significance level. Baseline characteristics are summarised overall and by trial arm as randomised, and as analysed in the primary effectiveness model for AUDIT at six months to assess the impact of attrition on the balance achieved across the randomised groups.

A total AUDIT score was only computed where all 10 component items were completed. The LDQ, SSQ and CORE-10 were considered complete with up to two missing values; missing values were replaced with the mean of the completed item scores. Substance subscale scores for the ASSIST were calculated where a response was provided for each question in that subscale, and a total ASSIST score derived from the sum of the seven subscales. The AUDIT, LDQ, SSQ and CORE-10 at six months were analysed separately using ANCOVA adjusting for the baseline score of the dependent variable and treatment group. An ANCOVA for ASSIST was not conducted due to insufficient observations and so the treatment groups were compared using a two-sample *t*-test. Outcomes at twelve months were compared between the two groups using *t*-tests.

In a sensitivity analysis, separate logistic regressions to predict response to the six month questionnaire were conducted for each trial arm, adjusting for the number of treatment sessions attended. The reciprocals of the resulting predicted proportions were then used as probability weights in the adjusted regressions for each outcome to allow for missing follow-up data in a similar method to that described and used in the ODIN trial (Dowrick et al., 2000).

Individual scale scores for the WAI were computed only where all the relevant items for that scale were complete; *t*-tests were used to compare each scale between the treatment groups.

### 2.8 Economic analysis

The analysis explored the cost of the interventions, the cost of health care and social services, and the cost of policing and criminal justice system, respectively. The intervention cost encompassed staff training cost and intervention delivery cost, which was recorded by the research team in the trial, accounting for staff time, premises, materials, other intervention expenses and overheads. The use of health care and social services cost was collected by self-report using a service use questionnaire in the baseline and follow-up surveys. Finally, participants were also asked in the same time about their contact with policing and the criminal justice system (court appearance and prison). A common set of national unit cost estimates was applied to the quantities recorded. The corresponding inflation rates were used where applicable (Curtis, 2013). All costs were presented in 2012/13 prices.

As per National Institute for Health and Care Excellence Guidance [NICE] (NICE, 2013), EQ-5D (EuroQol Group, 1990) responses were collected at baseline, six and twelve months follow-up to assess quality of life.

## 3 Results

**Fig. 1:**
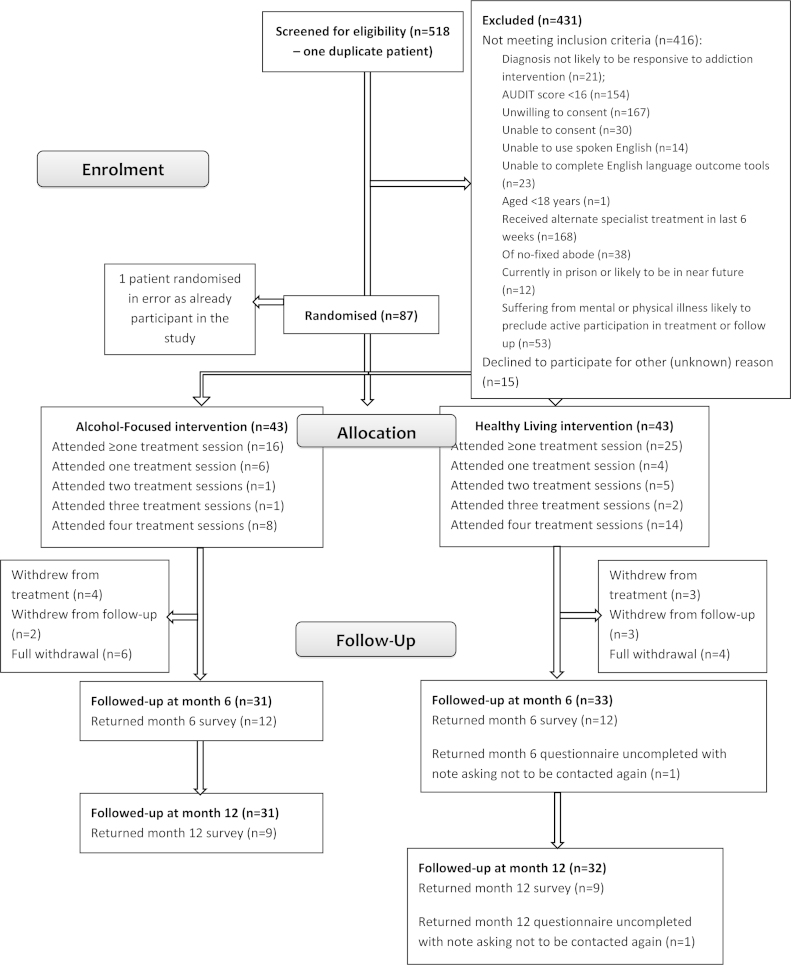
CONSORT diagram.

Overall, 517 patients were screened for eligibility between May, 2012 and December, 2012, of which 81% (*n* = 416) failed at least one eligibility criterion (Fig. 1). In all, 43 individuals were randomised to the AF arm and 43 to the HL arm. Most participants were male (*n* = 69, 80%) and the mean age was 46 years (SD 11.1, range 25 to 74) (Table 1).

### 3.1 Compliance

At least one treatment session was scheduled for 69 of the 86 (80%) randomised participants. A higher proportion of HL participants attended at least one treatment session (*n* = 25, 58%, compared to *n* = 16, 37%, for the AF group). The HL group also attended more sessions on average (*n* = 2) than the AF group (*n* = 1) and were more likely to attend all four treatment sessions (*n* = 14, 33% HL arm; *n* = 8, 19% AF arm).

### 3.2 Healthy living domains

In the HL group, drinking was the most commonly chosen health behaviour (*n* = 24), followed by diet (*n* = 13), exercise (*n* = 13), personal care (*n* = 6), smoking (*n* = 3), drugs (*n* = 1), and medication concordance (*n* = 1). The most popular combination of three domains was drinking, diet and exercise.

### 3.3 Attrition and questionnaire response rates

Ten patients withdrew from all aspects of the study (*n* = 4 HL, *n* = 6 AF); seven withdrew from treatment, but were willing to complete follow-up (*n* = 3 HL, *n* = 4 AF); and seven withdrew from follow-up, but may or may not have attended subsequent treatment sessions (*n* = 5 HL, *n* = 2 AF). Questionnaire response rates declined significantly at each time point: at six months, 25 questionnaires were returned (29%); and at twelve months, 19 questionnaires were returned (22%).

### 3.4 Clinical outcomes

#### 3.4.1 Six and 12 months

There was no evidence of a difference in AUDIT, ASSIST, LDQ, SSQ or CORE-10 score between the treatment groups at either months 6 or 12 (Table 2).

#### 3.4.2 Sensitivity analyses

The adjusted regression analyses were repeated using inverse probability weights to account for missing follow-up data. The following adjusted mean differences were observed:

• AUDIT: −10.0 (95% CI −14.4 to −5.6, *p* = 0.001)
• LDQ: 2.4 (95% CI −5.0 to 9.7, *p* = 0.50)
• SSQ: 6.0 (95% CI 1.7 to 10.4, *p* = 0.01)
• CORE-10: −10.1 (95% CI −14.7 to −5.6, *p* < 0.001)

The directions of the differences are the same as those observed in the un-weighted regression analyses although, barring the LDQ, the magnitudes are greater and the differences are statistically significant at the 5% level.

#### 3.4.3 Abstinence

At six months, eight participants (four in each arm) reported they had abstained from drinking alcohol in the previous six months; seven had attended all four treatment sessions and one (AF arm) had not attended any. At twelve months, five participants (HL *n* = 2; AF *n* = 3) reported abstinence in the previous six months; two in each arm had attended all four treatment sessions, and the third participant in the AF arm attended two.

#### 3.4.4 Working Alliance Inventory

Adjusted mean differences between the scale scores for the randomised arms were not statistically significant, except for the Bond scale on the patient WAI for session three (*p* = 0.02).

### 3.5 Health economic findings

The average intervention cost of the AF arm was lower than that of the HL arm (£32 per participant vs £60 per participant).

From baseline, the average use of health care and social services decreased in both arms at follow-up and was considerably lower in the AF arm than the HL arm (Table 3).

The mean cost of contact with policing and the criminal justice system also decreased at follow-up, with none observed in the HL arm at twelve months (Table 3).

Overall, the mean resource use in the AF arm was higher than the HL arm at baseline (£9773 vs £7639). However, costs reduced significantly in the AF arm at six months to £1158, and then remained at a similar level at twelve months (£1198), while in the HL arm it had a gradual decline (£6449 at six months, £3732 at twelve months) (Table 3).

All the national average unit costs adopted or estimated are shown in Table S1^1^.

The mean EQ-5D score in the HL arm was 0.45 (SD: 0.358) at baseline (*n* = 40), 0.55 (SD: 0.407) at six month (*n* = 10), and 0.63 (SD: 0.255) at twelve month (*n* = 8). The mean EQ-5D score in the AF arm was 0.47 (SD: 0.406) at baseline (*n* = 39), 0.77 (SD: 0.243) at six months (*n* = 11), and 0.66 (SD: 0.442) at twelve month (*n* = 7).

## 4 Discussion

This study aimed to examine the relative feasibility, acceptability and effectiveness of an AF intervention and an HL intervention for patients with alcohol-related hospital admissions who were identified, via screening, as problem drinkers. From a projected maximum of 222 trial participants, actual recruitment was 86. The number of people ineligible by virtue of already being in treatment or attending other services was higher than anticipated, which may reflect the relatively high AUDIT inclusion score of 16 or higher. Those who were eligible but declined were not required to give reasons and therefore details were not always available: however, of the 167 patients who declined to take part, sixteen (10%) stated that they were not willing to engage in treatment. Four others stated they wished to continue drinking or felt they had adequate help at home to address problems. This is a high rate of refusal when compared with previous studies, where the focus has been on delivering brief interventions in a hospital setting (Freyer-Adam et al., 2008; Holloway et al., 2007; Lock et al., 2006). The higher rate in the present study may therefore reflect reluctance for non-help seekers to take part in a trial that will involve a substantial intervention in a specialist addiction service.

There were notable but statistically insignificant differences in the engagement with the two interventions, with the HL group being more likely to attend treatment and attend more sessions. The fact that these differences occurred despite there being no noticeable differences in the WAI scores, suggests that it may have been the content of treatment that accounted for the different levels of engagement.

We found no evidence of a difference in AUDIT score between the treatment groups at six months. Due to the low recruitment and the extent of missing follow-up data, the study was ultimately underpowered to detect a clinically meaningful difference. Analyses are therefore exploratory and the implication of the results of the statistical and economic analysis should be interpreted with caution. In a small trial like this one, low frequency high tariff costs are unlikely to be evenly distributed by trial arms and can fall by chance into one group or another. For instance, all the longer hospital stays occurred in the HL arm, resulting in consistently higher cost, and possibly the consistently lower EQ-5D score, in this arm. Similarly, the numbers of arrests, cautions or on-the-spot fines, and Magistrates’ Court and Crown Court appearances in the AF arm were all higher than in the HL arm. Although there is no sufficient data to conclude a significant impact of either intervention on post-treatment health care and social resources use, the findings shed a light on potential societal gains in the event of effective treatment for this patient group.

Participants were sometimes too unwell to be seen soon after first contact, or they were lost to contact once discharged from hospital. Many declined to be seen for any reason other than that for which they had been admitted. There had initially been some discussion in the research team about whether the first appointment should occur in the hospital before the patient was discharged. This would have allowed for all participants to have received at least one treatment session and therefore an opportunity for a therapeutic relationship to be developed. Suitable accommodation for this purpose was generally unavailable in the hospitals included in this trial, but this approach could possibly be considered in future trials. Although multiple attempts were made, via mail and telephone, to encourage response, these some participants either ignored them or became unreachable due to changes in provided contact number(s) or address. Additional follow-up strategies for follow-up have been used in other studies (Owens et al., 2011), including appointment reminders, contacting a participant's General Practitioner (GP), offering a range of follow-up options and finally, if a participant cannot be contacted or fails to respond, visiting their home. While these methods are labour intensive options, they are likely to increase follow-up rates and should be considered in future studies.

The findings of this trial have therefore illustrated some of the challenges of working with this particular population. Studies aiming to recruit patients in hospital who are drinking at harmful or dependent levels need to take account of the fact that many will already be in treatment or will have recently been in treatment. Such studies also need to allow for the reluctance of many to take part in a trial which will involve treatment at a specialist addiction service. This may due to a refusal among some to accept that even high levels of drinking are damaging their health. It may also involve a reluctance to attend an addiction service, with the concomitant recognition that they ‘have a problem’ and the associated potential for stigmatisation (Lloyd, 2013). Lastly, the high rate of loss to follow up demonstrates the need for particularly intensive follow-up efforts with this group.

### 4.1 Strengths and limitations

Healthy lifestyle approaches do not appear to have received much emphasis in the alcohol treatment literature (Brown et al., 2009), despite their potential (Marlatt and Gordon, 1985; Marlatt and Witkiewitz, 2005). This study has attempted to address this by investigating whether a healthy lifestyle approach may be more acceptable in a non-help seeking population of problem drinkers than an explicit emphasis on changing drinking behaviour alone.

This study was preceded by a training project which aimed to address the challenge of getting hospital staff on board and was successful in improving the existing shared working between hospital staff and the specialist in-reach service, especially regarding the identification of problem drinkers on the wards, and the accessibility and acceptance of the specialist team.

This study was hampered by low rates of engagement, treatment uptake and follow-up. The reluctance of this patient group to attend treatment sessions and complete postal follow-up questionnaires has highlighted the need for other methods to be used. In addition, there is a possibility that on discharge and away from the hospital environment, any concerns regarding their drinking levels became diminished and no longer of any importance.

### 4.2 Conclusions

The results of this study demonstrate a potential to engage patients drinking at harmful or dependent levels in a healthy living intervention, which in this study, was at least as acceptable as an alcohol-focused intervention. However, perhaps the key conclusions from this trial relate to the difficulty of engaging with this group: both in regard to treatment and research. Despite high levels of drinking and drinking-related harm, many in this group still show a reluctance to participate in a study that will involve them in specialist treatment for their drinking problem. Given the high cost of this group to the NHS, we need to find better ways of helping these patients recognise the harms associated with their drinking and overcome the evident barriers to their engagement with specialist treatment.

## Author disclosures

### Role of funding source

This article presents independent research by the Addiction Research in Acute Settings (ARiAS) theme of the National Institute for Health Research Collaboration for Leadership in Applied Health Research and Care for Leeds, York and Bradford (NIHR CLAHRC LYB), a pilot which ended in 2013. Further details about the new NIHR CLAHRC Yorkshire and Humber can be found at www.clahrc-yh.nihr.ac.uk. The views and opinions expressed are those of the authors, and not necessarily those of the NHS, the NIHR or the Department of Health.

## Contributors

The ARiAS research team had the original idea for this study and all authors contributed to the final design. CG, DR and GT secured the CLAHRC funding for the ARiAS theme, which included this pilot study. DR was the study Chief Investigator. GT and DR were responsible for the clinical management of the study. CL and CG were responsible for the academic research aspects of the study, to which GT and DR contributed. HC was responsible for study co-ordination and overseeing data collection at the clinical site. JW had overall responsibility for the study management supported by NM at the coordinating centre. VD and SP were responsible for the statistical and health economic design respectively and CF and JL conducted the analyses. PT was responsible for the design, conduct and analysis of the qualitative work. CF, JW and JL wrote the first draft of the paper and all authors contributed to the editing. All authors read and approved the final manuscript.

## Conflict of interest statement

No conflict declared.

## Figures and Tables

**Table 1:** Baseline participant characteristics and treatment preference by trial arm as randomised and as analysed in the primary effectiveness analysis.

	Healthy living (*n* = 43)	Alcohol-focused (*n* = 43)	Total (*n* = 86)	Healthy living (*n* = 8)	Alcohol-focused (*n* = 7)	Total (*n* = 15)
Age (years)						
Mean (SD)	45.9 (12.5)	47.5 (9.5)	46.7 (11.1)	42.8 (5.1)	48.1 (8.9)	45.3 (7.4)
Gender, *n* (%)						
Male, *n* (%)	35 (81.4)	34 (79.1)	69 (80.2)	6 (75.0)	6 (85.7)	12 (80.0)
Smoking status, *n* (%)						
Ex-smoker	10 (25.0)	3 (7.5)	13 (16.3)	1 (14.3)	1 (14.3)	2 (14.3)
Current smoker	27 (67.5)	34 (85.0)	61 (76.3)	5 (71.4)	3 (42.9)	8 (57.1)
Never smoked	3 (7.5)	3 (7.5)	6 (7.5)	1 (14.3)	3 (42.9)	4 (28.6)
Employment, *n* (%)						
In employment	8 (18.6)	8 (18.6)	16 (18.6)	2 (25.0)	4 (57.1)	6 (40.0)
Days off sick in past 6 months						
Median (min, max)	3 (0, 25)	5 (0, 180)	3 (0, 180)	7 (7, 7)	2 (0, 20)	4.5 (0, 20)
Marital status, *n* (%)						
Single	29 (74.4)	28 (70.0)	57 (72.1)	6 (85.7)	4 (57.2)	10 (68.7)
Married	6 (15.1)	6 (15.0)	12 (15.2)	1 (14.3)	1 (14.3)	2 (14.3)
Co-habiting	4 (10.3)	6 (15.0)	10 (12.7)	0 (0.0)	2 (28.6)	2 (14.3)
Accommodation, *n* (%)						
Owner occupied	7 (17.5)	10 (25.0)	17 (21.3)	2 (28.6)	2 (28.6)	4 (28.6)
Other	33 (82.5)	30 (75.0)	63 (78.8)	5 (71.4)	5 (71.4)	10 (71.4)
Education post age 16, *n* (%)	17 (42.5)	15 (37.5)	32 (40.0)	4 (57.1)	2 (28.6)	6 (42.9)
Educated to degree level, *n* (%)	6 (15.0)	8 (21.1)	14 (18.0)	0 (0.0)	1 (14.3)	1 (7.1)
No. of medications						
0	15 (37.5)	9 (22.5)	24 (30.0)	3 (42.9)	2 (28.6)	5 (35.7)
1	1 (2.5)	3 (7.5)	4 (5.0)	0 (0.0)	1 (14.3)	1 (7.1)
2	7 (17.5)	3 (7.5)	10 (12.5)	0 (0.0)	0 (0.0)	0 (0.0)
3+	17 (42.5)	25 (62.5)	42 (52.5)	4 (57.1)	4 (57.1)	8 (57.1)
Treatment preference, *n* (%)						
The alcohol-focused intervention	15 (37.5)	11 (30.6)	26 (34.2)	2 (28.6)	2 (33.3)	4 (30.8)
The healthy living intervention	11 (27.5)	7 (19.4)	18 (23.7)	1 (14.3)	0 (0.0)	1 (7.7)
Neither	0 (0.0)	0 (0.0)	0 (0.0)	0 (0.0)	0 (0.0)	0 (0.0)
No preference	14 (35.0)	18 (50)	32 (42.1)	4 (57.1)	4 (66.7)	8 (61.5)

**Table 2:** Summary of outcome measures at each time point and mean differences (HL-AF).

Outcome measure	Healthy living (*n* = 43)	Alcohol-focused (*n* = 43)	Overall (*n* = 86)	Mean difference* (95% CI) *p*-value
Audit				
Baseline	*N* = 43	*N* = 42	*N* = 85	
Mean (SD)	31.5 (5.9)	31.5 (6.1)	31.5 (6.0)
Month 6	*N* = 8	*N* = 8	*N* = 16	5.0 (−2.7, 12.7) *p* = 0.19
Mean (SD)	33.4 (5.4)	24.6 (7.2)	29 (7.7)
Month 12	*N* = 6	*N* = 5	*N* = 11	5.0 (−13.6, 23.6) *p* = 0.56
Mean (SD)	25 (11.5)	20 (15.7)	22.7 (13.1)
Assist total				
Baseline	*N* = 15^^^	*N* = 19	*N* = 34	
Mean (SD)	13.4 (15.6)	17.1 (24.1)	15.5 (20.6)
Month 6	*N* = 7	*N* = 3	*N* = 10	0.5 (−12.2. 13.1) *p* = 0.93
Mean (SD)	5.1 (8.9)	4.7 (4.2)	5 (7.5)
Month 12	*N* = 3	*N* = 3	*N* = 6	8.0 (−16.8, 32.8) *p* = 0.42
Mean (SD)	14.3 (12.9)	6.3 (8.5)	10.3 (10.7)
LDQ				
Baseline	*N* = 40	*N* = 40	*N* = 80	
Mean (SD)	20.3 (7.6)	17.8 (7.2)	19.1 (7.4)
Month 6	*N* = 13	*N* = 11	*N* = 24	−4.7 (−14.5, 5.0) *p* = 0.32
Mean (SD)	12.5 (11.2)	12.0 (11.4)	12.3 (11.1)
Month 12	*N* = 9	*N* = 9	*N* = 18	1.0 (−10.5, 12.5) *p* = 0.86
Mean (SD)	9.7 (11.0)	8.7 (12.0)	9.2 (11.2)
SSQ				
Baseline	*N* = 40	*N* = 40	*N* = 80	
Mean (SD)	13.5 (6.0)	14.2 (5.2)	13.8 (5.6)
Month 6	*N* = 12	*N* = 11	*N* = 23	−4.9 (−9.7, 0.02) *p* = 0.05
Mean (SD)	9.8 (6.2)	15.0 (6.7)	12.3 (6.8)
Month 12	*N* = 10	*N* = 9	*N* = 19	−3.2 (−8.9, 2.4) *p* = 0.24
Mean (SD)	13.6 (6.6)	16.8 (4.7)	15.1 (5.9)
CORE-10				
Baseline	*N* = 39	*N* = 40	*N* = 79	
Mean (SD)	24.4 (7.8)	21.0 (7.6)	22.7 (7.8)
Month 6	*N* = 13	*N* = 11	*N* = 24	2.0 (−7.8, 11.8) *p* = 0.68
Mean (SD)	18.5 (10.1)	13.5 (9.8)	16.2 (10.0)
Month 12	*N* = 9	*N* = 9	*N* = 18	7.6 (−2.0, 17.2) *p* = 0.11
Mean (SD)	19.6 (10.2)	12.0 (9.1)	15.8 (10.1)

AUDIT—Alcohol Use Disorders Identification Test, ASSIST—Alcohol, Smoking and Substance Involvement Screening Test, LDQ—Leeds Dependence Questionnaire, SSQ—Social Satisfaction Questionnaire, CORE-10—10-item Clinical Outcomes in Routine Evaluation.

* All analyses of month 6 outcomes adjusted for baseline except ASSIST, all month 12 comparisons unadjusted. ^^^ Indicates the number who said they had ever used a drug from this class.

**Table 3:** Mean cost of different costing components in the previous six months (SD), by intervention group.

	Intervention group	
	Alcohol-focused	Healthy living
Baseline	*N* = 39	*N* = 38
Health care and social services	£4831 (£5145)	£5525 (£7140)
Police and criminal justice system	£5066 (£13,476)	£2114 (£5842)
Total	£9773 (£16,676)	£7639 (£8472)
Month 6	*N* = 11	*N* = 11
Health care and social services	£1,100 (£1738)	£5673 (£5426)
Police and criminal justice system	£64 (£202)	£826 (£2741)
Total	£1158 (£1744)	£6499 (£5852)
Month 12	*N* = 10	*N* = 9
Health care and social services	£887 (£1010)	£3732 (£6455)
Police and criminal justice system	£311 (£932)	£0 (£0)
Total	£1198 (£1756)	£3732 (£6455)

## Footnotes

1 Supplementary material can be found by accessing the online version of this paper at http://dx.doi.org and by entering doi:10.1016/j.drugalcdep.2015.06.030.
