# Supplementary material for: ADAPTA: A pilot randomised controlled trial of an alcohol-focused intervention versus a healthy living intervention for problem drinkers identified in a general hospital setting
Source: Drug Alcohol Depend. 2015 Sep 1;154:117–24. doi: 10.1016/j.drugalcdep.2015.06.030 (PMC4545229; doi:10.1016/j.drugalcdep.2015.06.030)
Supplement: Supplementary file 1 [file mmc1.docx]

**Supplementary Material for the article:**

**ADAPTA: a pilot randomised controlled trial of an alcohol-focused intervention versus a healthy living intervention for problem drinkers identified in a general hospital setting.**

**Judith M Watson^1^ (jude.watson@york.ac.uk)**

**Caroline Fairhurst^1^ (caroline.fairhurst@york.ac.uk)**

**Jinshuo Li^1^ (jinshuo.li@york.ac.uk)**

**Gillian Tober^2^ (gillian.tober@nhs.net)***

**Helen Crosby^2^ (helen.crosby@nhs.net)**

**Charlie Lloyd^1^ (charlie.lloyd@york.ac.uk)**

**Christine Godfrey^1^ (christine.godfrey@york.ac.uk)**

**Noreen D. Mdege^1^ (noreen.mdege@york.ac.uk)**

**Veronica Dale^1^ (veronica.dale@york.ac.uk)**

**Paul Toner^1^ (paul.toner@york.ac.uk)**

**Steve Parrott^1^ (steve.parrott@york.ac.uk)**

**Duncan Raistrick^2^ (d.raistrick@nhs.net)**

**On behalf of the ARiAS Research Group, NIHR CLAHRC for Leeds, York and Bradford**

***1 University of York, Department of Health Sciences, Heslington, York, United Kingdom YO10 5DD***

***2 Leeds Addiction Unit, 19 Springfield Mount, Leeds, United Kingdom LS2 9NG***

***Corresponding author – Tel: +44 (0) 113 855 9558 Fax: +44 (0) 113 295 2770**

**Email: gillian.tober@nhs.net**

**This material supplements, but does not replace, the peer-reviewed article in**

***Drug and Alcohol Dependence.***

*Table S1. Unit cost of health care and social services, police activity and criminal justice system*

| Resources | Unit cost (2012/13) | Sources |
| --- | --- | --- |
| Accident and Emergency | £115 / Visit | Department of Health 2013 |
| Hospital stay | £542 / Day | Department of Health 2013 |
| Hospital outpatient | £108 / Visit | Department of Health 2013 |
| Day case | £693 / Case | Department of Health 2013 |
| Emergency ambulance | £96 / Ride | Department of Health 2013 |
| Patient transport service | £34 / Ride | £32 in 2009/10, inflated with HCHS index to 2012/13 price (Department of Health 2011; 2013) |
| Doctor at GP practice | £45 / Visit | Curtis, 2013 |
| Doctor home visit | £114 / Visit | Curtis, 2013 |
| Nurse at GP practice | £11 / Visit | Recalculated based on the average duration of visits (Curtis, 2013) |
| Nurse home visit | £78 / Visit | Recalculated based on the average number of visits per day (Curtis, 2013) |
| Prescription | £17 / Prescription | Calculated based on the average number of item per prescription 2007-2011 and average cost per prescription item 2012 (HSCIC, 2013; NHS Business Services Authority, 2012) |
| Crisis team | £188 / Meeting | £184 in 2011/12 price, inflated with HCHS index to 2012/13 price (Curtis, 2012; 2013) |
| Social worker home visit | £178 / Visit | Assuming a one-hour contact per visit and 1: 0.45 ratio of direct to indirect time on home visits as community nurse (Curtis, 2013) |
| Social worker in office visit | £159 / Visit | Assuming a one-hour contact per visit (Curtis, 2013) |
| Arrest, caution or penalty disorder notice (PND) | £2,796 / Incident | (Curtis, 2013; Ministry of Justice, 2013; HM Treasury, 2013; Field, 1997) |
| Magistrate’s court appearance | £639 / Appearance | (HM Courts & Tribunal Services, 2013; Legal Services Commission, 2013; Crown Prosecution Service, 2013; Phillips and Brown, 1998) (HMCTS Governance and Assurance Data and Information Disclosures, Ministry of Justice, Freedom of Information Request by e-mail, 9^th^ April 2014) (Crown Prosecution Service, Freedom of Information Request by e-mail, 7^th^ April 2014) |
| Crown Court appearance | £11,586 / Appearance |  |
| Prison days | £111 / Day | Curtis, 2013; Brookes *et al,* 2013 |

**REFERENCES**

Brookes, N., Barrett, B., Netten, A., Knapp, E., 2013. Unit Costs in Criminal Justice(UCCJ). London School of Economics, University of Kent..

Crown Prosecution Service, 2013. Crown Prosecution Service Annual Report and Accounts 2012-13, hc31. The Stationary Office, London.

Curtis, L., 2012. Unit Costs of Health and Social Care 2012. Personal Social Services Research Unit, University of Kent.

Department of Health., 2011. Reference Costs 2009-10 Publication. Department of Health, London.

Department of Health, 2013. Reference costs 2012-13. Department of Health, London.

Fairhurst, C., Böhnke, J.R., Gabe, R., Croudace, T., Tober, G., Raistrick, D., 2014. Factor analysis of treatment outcomes from a UK specialist addiction service: relationship between the Leeds Dependence Questionnaire, Social Satisfaction Questionnaire and 10-item Clinical Outcomes in Routine Evaluation. Drug Alcohol Rev**.** 33, 643-50.

Field, S., 1997. Flows and Costs in the Criminal Process. Home Office, London.

HM Courts & Tribunals Service, 2013. HM Courts & Tribunals Service Annual Report and Accounts 2012-13, hc239, The Stationary Office, London.

HM Treasury, 2013. Public Expenditure: Statistical Analyses 2013, cm8663. The Stationary Office, London.

HSCIC (Health and Social Care Information Centre Prescribing and Primary Care Services NHS England)., 2013. Prescription Cost Analysis England 2012, Health and Social Care Information Centre, Leeds.

Legal Services Commission., 2013. Legal Services Commission Annual Report and Accounts 2012-13, hc337. The Stationary Office, London.

Ministry of Justice, 2013. Statistics on Race and the Criminal Justice System 2012.Ministry of Justice, London.

NHS Business Services Authority, 2012. PD1. NHS Business Services Authority,London.

Phillips, C., Brown, D., 1998. Entry into the Criminal Justice System: a Survey ofPolice Arrests and their Outcomes. Home Office Research Study. Home Office,London, pp. 185
